# Genotype calling in tetraploid species from bi-allelic marker data using mixture models

**DOI:** 10.1186/1471-2105-12-172

**Published:** 2011-05-19

**Authors:** Roeland E Voorrips, Gerrit Gort, Ben Vosman

**Affiliations:** 1 Plant Breeding Department, Wageningen University and Research Centre, Wageningen, The Netherlands; 2 Biometris, Wageningen University and Research Centre, Wageningen, The Netherlands; 3 Centre for BioSystems Genomics, P.O. Box 98, 6700 AB Wageningen, The Netherlands

## Abstract

**Background:**

Automated genotype calling in tetraploid species was until recently not possible, which hampered genetic analysis. Modern genotyping assays often produce two signals, one for each allele of a bi-allelic marker. While ample software is available to obtain genotypes (homozygous for either allele, or heterozygous) for diploid species from these signals, such software is not available for tetraploid species which may be scored as five alternative genotypes (aaaa, baaa, bbaa, bbba and bbbb; nulliplex to quadruplex).

**Results:**

We present a novel algorithm, implemented in the R package fitTetra, to assign genotypes for bi-allelic markers to tetraploid samples from genotyping assays that produce intensity signals for both alleles. The algorithm is based on the fitting of several mixture models with five components, one for each of the five possible genotypes. The models have different numbers of parameters specifying the relation between the five component means, and some of them impose a constraint on the mixing proportions to conform to Hardy-Weinberg equilibrium (HWE) ratios. The software rejects markers that do not allow a reliable genotyping for the majority of the samples, and it assigns a missing score to samples that cannot be scored into one of the five possible genotypes with sufficient confidence.

**Conclusions:**

We have validated the software with data of a collection of 224 potato varieties assayed with an Illumina GoldenGate™ 384 SNP array and shown that all SNPs with informative ratio distributions are fitted. Almost all fitted models appear to be correct based on visual inspection and comparison with diploid samples. When the collection of potato varieties is analyzed as if it were a population, almost all markers seem to be in Hardy-Weinberg equilibrium. The R package fitTetra is freely available under the GNU Public License from http://www.plantbreeding.wur.nl/UK/software_fitTetra.html and as Additional files with this article.

## Background

Several important agronomic and horticultural crops are tetraploids, including potato (*Solanum tuberosum*), leek (*Allium porrum*) and alfalfa (*Medicago sativa*). In such species marker alleles can be present in different dosages, ranging from 0 (nulliplex) to 4 (quadruplex). Differences in allele dosage may result in differences in the RNA levels of a particular allele and in phenotypic differences [1]. Therefore it is important to be able to exactly determine the allele dosage.

In species with tetrasomic inheritance like autotetraploids the four copies of each chromosome may recombine with each other, showing no or little preferential pairing [2]. In contrast, in allotetraploids, also termed amphidiploids, in effect there are two different genomes that show little or no recombination.

Genetic studies in species with tetrasomic inheritance have lagged behind those in diploids because segregation patterns are more complex. The exception is where one parent of a cross contains an allele in single dose (simplex, abbb) and the other parent is homozygous (nulliplex, bbbb); in this case segregation is exactly as in a diploid heterozygous × homozygous cross. While software has been developed for linkage analysis including duplex marker segregation and multi-allelic markers [3], genetic studies in tetraploids rely mostly on simplex × nulliplex marker segregation, or on simplifying assumptions regarding chromosomal pairing [4]. Still, the construction of linkage maps based on such simplex × nulliplex markers is problematic because most of the markers will be in repulsion phase (two tetraploid cross parents have 8 homologs, so only one in 8 pairs of simplex × nulliplex markers are in linkage phase), meaning that genetic distances can be assessed only with low precision.

With the advent of high-throughput SNP genotyping technologies the number of available markers is increasing dramatically. To take full advantage of these technologies it is desirable to make use of all segregating markers in a population, not just the simplex × nulliplex markers. Also for association mapping in collections of genotypes the use of as many markers as possible would be useful [5,6]. This requires the ability to score allele dosage, in contrast to just presence or absence of an allele, like in the case of simplex × nulliplex markers.

In the past highly polymorphic SSR markers have been used to study segregation of alleles in polyploid species [7,8] and a general method, MAC-PR (microsatellite DNA allele counting - peak ratios) was developed [9]. In MAC-PR ratios between microsatellite peak areas are used to deduce the allelic configuration of a polyploid plant. The current technologies for SNP genotyping [10], including Illumina GoldenGateTM [11] and Infinium array [12,13] typically generate two signals, one for each of two alleles at a marker locus. In principle, these signals are proportional with the allele dosage, e.g. one of five classes from nulliplex to quadruplex in a tetraploid species. However, in real life both signals are generally continuous, making it more difficult to assign a sample to a specific class. One approach to convert the continuous signal scores to discrete genotype classes is to apply a clustering algorithm to the two-dimensional signal data. This is the approach used e.g. by Illumina's proprietary GenomeStudio software http://www.illumina.com/software/genomestudio_software.ilmn for diploid samples. In the diploid case only three genotype classes are possible, and assigning a genotype class to each cluster is not too difficult, even if one of the classes does not occur. However with tetraploid samples the problem is more complex. There are five instead of three possible genotype classes, which makes the cluster separation more difficult. Also, possibly occurring null alleles (alleles that are not recognized by the assay and hence do not generate a signal) may produce one or two additional clusters, in contrast to the diploid case. Finally, in a clustering approach the issue arises how to match the clusters to the different genotypes; in a tetraploid the number of clusters and genotypes is larger which complicates this matching, especially if less than five clusters are detected. Very recently, a software package, *beadarrayMSV *became available that is able to analyse Illumina BeadArrays in the partly duplicated genomes and uses a clustering approach to discriminate five possible genotypes [14]; this is discussed in more detail below.

We present here an alternative approach based on mixture models. Mixture models have been used in the codominant scoring of AFLP band intensities for diploid species [15,16], and specifically in collections of genotypes [17]. Our approach is based on the allele signal ratio, i.e. the fraction of the *a *signal in the total signal. We fitted a mixture of five normal distributions to the allele signal ratios, with each distribution representing one of the five possible genotype classes. The means of the five distributions are constrained by the corresponding allele ratios (see Implementation - The mixture model). Because we model the component means explicitly as a function of the allele ratios, the assignment of components to genotype classes is in our case automatic. Furthermore, the relation between allele ratios and means of the distributions helps to identify each distribution, even when the distributions overlap considerably. These advantages compared with a clustering approach are well worth the considerable extra computation time required.

## Implementation

### The mixture model

We fit mixture models to the response signals to classify the markers into one of five genotype classes, corresponding to the five possible allele dosages in tetraploids. This type of classification is often called model-based clustering, because a statistical model is used for the responses. We describe the model here.

Let the pair *s_a_*and *s_b_*represent the measured a and b allele signal strengths for an individual. We analyze the fraction *s_a_*/(*s_a_*+ *s_b_*). As it is advantageous to have a homoscedastic response in the mixture model, and the calculated fraction shows variance heterogeneity with smaller variation for fractions closer to 0 and 1, we take the arcsine-square root (*asr*) transformed fraction  to stabilize the variance.

For the transformed fraction *y *a normal (or Gaussian) mixture model [18] is fitted:

with *f_j_*the density of a normal distribution with mean *μ_j_*and common standard deviation *σ*. The mixing probabilities *π_j_*are the prior probabilities of a marker to have allele dosage *j*, with Σ*_j_π_j_*= 1. In the model described above, five components are specified for the five allele dosages (0,..,4), but in other situations less or more components may be needed. In case of five components, ten model parameters have to be estimated: five means *μ_j_*(for the mean responses of the five allele dosages), one standard deviation *σ *(measuring the common spread of individual responses with the same allele dosage), and four probabilities *π_j_*(measuring the fraction of individuals having the *j*^th ^allele dosage), with the fifth probability following from the other four.

One of the principles in statistical modelling is parsimony: remove redundant model parameters to improve stability and interpretability of results. Here it may be beneficial to put constraints on two groups of parameters:

1) Constraints on *π_j_*according to Hardy-Weinberg equilibrium (HWE). If the allele dosages are in HWE, a single parameter *p*, representing the allele frequency in the population, suffices instead of four probabilities *π_j_*. The constraints are *π*_1 _= *p*^4^, *π*_2 _= 4*p*^3^(1-*p*), *π*_3 _= 6*p*^2^(1-*p*)^2^, *π*_4 _= 4*p*(1-*p*)^3^, *π*_5 _= (1-*p*)^4^.

2) Constraints on *μ_j_*, by incorporating an assumed relationship between allele dosage and signal strength. We first assume that the signal strengths *s_a_*and *s_b_*depend linearly on the allele dosage: with *x *the dosage of allele a, and 4-*x *the dosage of allele b, the model states for the mean signal strengths of *s_a_*and *s_b_* where *a*_0 _and *b*_0 _are the background signal strength for alleles a and b. The fraction  contains a superfluous parameter, and can be simplified into model 1:(1)

with *c*_1 _= *a*_0 _/*a*_1_, *c*_2 _= *b*_0 _/*a*_1_, and *r *= *b*_1 _/*a*_1_. Hence, parameters *c*_1 _and *c*_2 _are proportional to the background signals, and *r *is the ratio of sensitivities of the a and b signal strengths to the allele dosages.

If the a and b background signal strengths are equal, a common parameter *c *= *c*_1 _= *c*_2 _can be used to arrive at model 2:(2)

The assumption of a linear relationship between signal strength and allele dosage may be too restrictive. Therefore, the model for the individual signal strengths is extended into  assuming equal curvature for both signals, rendering the third model(3)

with *d *= *a*_2 _/*a*_1_.

Model 3 may be simplified into model 4, by equating the background signal strengths:(4)

Models (1) - (4) are formulated for the fraction of means of signal strengths. However, as the response is the *asr-*transformed variable *y*, the models need to be transformed as well. The transformed model (1) for the expectation of *y *is *μ_y_*= *asr*((*c*_1 _+ *x*)/(*c*_1 _+ *x *+ *c*_2 _+ *r *(4 - *x*))), and likewise for the other three models.

There are two minor complications with the models:

• The models 1-4 are developed for the fraction of expected signal strengths *E*(*s_a_*)/(E(*s_a_*) + E(*s_b_*)), but we analyze the fraction *s_a_*/(*s_a_*+ *s_b_*), amounting to a model for the expected fraction *E*(*s_a_*/(*s_a_*+ *s_b_*)). However, the expectation of a fraction and the fraction of expectations are approximately, but not exactly, equal.

• Transformation bias. We analyze the *asr*-transformed ratio of intensities *y *= *asr*(*s_a_*/(*s_a_*+ *s_b_*)), amounting to a model for the expectation *E*(*y*). This expectation is approximately, but not exactly, equal to *asr*(*E*(*y*)).

Summarizing, two approximations are employed: *E*(*asr*(*s_a_*/(*s_a_*+ *s_b_*))) ≈ *asr*(*E*(*s_a_*/(*s_a_*+ *s_b_*))) *≈ asr*(*E*(*s_a_*)/(*E*(*s_a_*) + *E*(*s_b_*))).

To compare different models, e.g. the unconstrained and HWE-constrained model, -2log-likelihoods (-2LL) may be compared, with by definition a smaller -2LL for the unconstrained (larger) model. To balance model fit and increased model complexity, we use the Bayesian Information Criterion (BIC), which adds a penalty to the -2LL based on the number of parameters *k *in the model (and *n *the number of individuals): BIC = -2LL+*k *ln(*n*) [19].

The different mixture models are fitted to the transformed fractions using maximum likelihood (ML). The EM-algorithm is used to find the ML-estimates [20]. The EM-algorithm needs starting values of the parameters. Next, E- and M-steps are iterated. In the E-step, given the current parameter values, the posterior probabilities of an individual to have each of five allele dosages are calculated, followed by the M-step, in which the mixture probabilities *π_j_*are estimated, and *μ_j_*and *σ *by weighted non-linear least squares. The fitting is done using R [21]. For a more elaborate description of mixture models for marker genotyping and the EM-algorithm, see [17].

### Model and marker selection

The selection of a suitable mixture model for a given marker is the result of a multi-step process that has been developed empirically.

Before starting the model selection itself, unreliable observations should be removed. In the case of an Illumina GoldenGate assay we removed all observations with a total signal intensity less than 3200 (see Data sets for the rationale for this threshold).

In the first step, eight different mixture models are fitted. Each model consists of 5 component distributions. The means of the component distributions are constrained by the five possible allele ratios, using one parameter for the ratio of intrinsic signal strength for both alleles, and additionally one or two parameters for signal background, and no or one coefficient for a quadratic term in the signal response (Equations 1-4). This results in four possible models for the means of the component distributions. Each of these models is combined with two models for the mixing proportions: (a) the mixing proportions are not constrained, or (b) the mixing proportions are constrained to Hardy-Weinberg equilibrium (HWE) ratios. The HWE restriction often helps in identifying the peaks, even if the actual ratios depart slightly from the HWE. As the EM algorithm does not always find the global maximum from a given start configuration of parameter values, the EM algorithm for these eight models is started with two different configurations of means: one where the five original means are derived from a hierarchical clustering of the signal ratios, and one where they are set at equidistant positions on the transformed scale from 0.142 to 1.429 (corresponding to 0.02 and 0.98 on the original scale).

The BIC of the 16 results are compared and the result with the minimum BIC is selected. Using the selected model, for every sample the probabilities of belonging to each of the five distributions are calculated. Only if the maximum probability is above a certain threshold (by default 0.99) the corresponding genotype class is assigned to the sample. This threshold affects the reliability of the genotype scores; a high threshold (such as the default) results in a high reliability but in less called genotypes; and if the percentage of called genotypes drops below a specified level (see below) the SNP is not scored at all.

If the difference in response between the two allelic signals is large (parameter *r *is much smaller or larger than 1), a wide gap occurs between the nulliplex or quadruplex peak and the next peak, while the other four peaks are closely spaced. In such cases it may happen that the EM algorithm does not find the optimal fit but instead fits the simplex or triplex peak in the wide gap. In order to detect and correct such mis-fits, a second step tests whether the simplex or triplex peaks have a lower mixing proportion or a smaller number of samples assigned than peaks at both sides. If this is the case, the eight models are fitted again with a third starting configuration for the distribution means: if the triplex peak appears to be fitted in the gap the means of the duplex, simplex and nulliplex peaks are reassigned to the triplex, duplex and simplex peak; for the nulliplex peak a new mean halfway between the (new) simplex mean and 0.0 is assigned. A similar rearrangement is made if the simplex peak appears to be fitted in the gap. Using this new starting configuration of the means the EM algorithm for the eight models is run again.

For each of the fitted models a check is done if a lower peak occurs between higher peaks. Neither in a cross progeny nor in a population in Hardy-Weinberg equilibrium such a pattern is expected. Therefore, by default the algorithm includes a third step which rejects all fitted solutions where such a pattern occurs; however, this check can be disabled. If in all fitted models lower peaks occur between higher peaks or if this check is disabled, no solutions are rejected in this step.

After these initial steps, the fitted model with the lowest BIC among the non-rejected solutions is selected. Again for every sample the probabilities of belonging to each of the five distributions are calculated and genotypes are assigned using the same criterion as in step 1.

In the final step, markers can be rejected based on several additional criteria. If less than a minimum fraction (by default 60%) of the samples are assigned a genotype this indicates an unclear peak pattern. This parameter interacts with the parameter specifying the minimum probability level required for assigning a genotype as described above. Also a peak variance above a certain threshold (by default 0.1 on the transformed scale) causes the marker to be rejected; again this filters against unclear peak patterns. This parameter may be decreased when the general noise level of the well-performing assays is low. A third criterion for marker rejection is when more than a maximum fraction (by default 85%) of the assigned samples are in the same peak. This parameter may be increased for data sets with more samples, as long as there are sufficient samples outside the main peak for reliable fitting of the remaining components of the mixture distribution.

It is recommended to try out some different values of the parameters based on the guidelines above and inspect the results for a subset of the markers, before selecting the values to apply to the full dataset.

### The program

**Figure 1:**
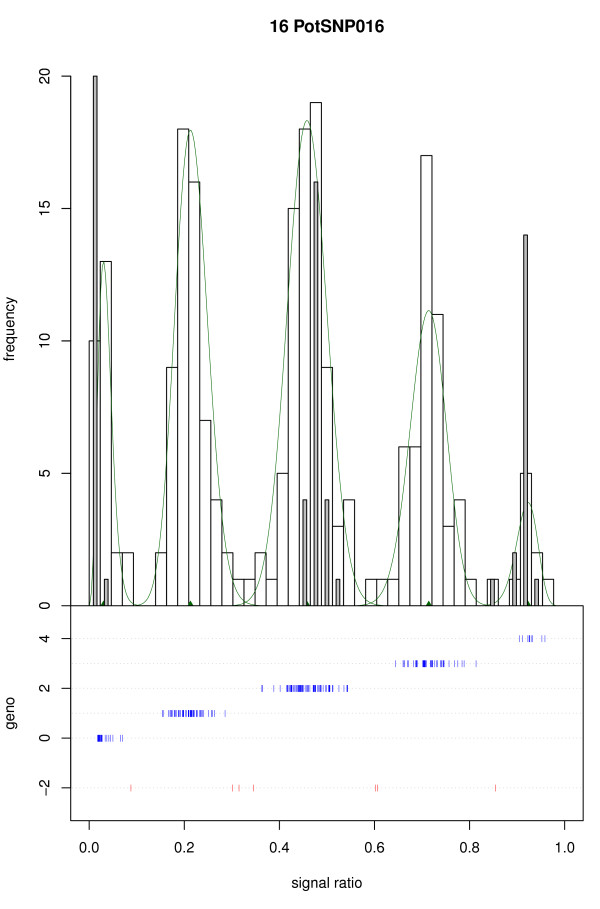
**Typical graphical output of fitTetra**. Upper panel: histogram of the signal ratios: allele a/(allele a + allele b) of a set of tetraploid potato varieties (white bars) and a diploid cross progeny (gray bars) for marker PotSNP016. The model fitted to the tetraploid varieties is indicated (green line). Lower panel: the genotype (0 to 4 for nulliplex to quadruplex) assigned to the tetraploid samples in relation to the signal ratios. Unassigned samples are shown at the bottom in red. The diploid samples coincide with the nulliplex, duplex and quadruplex peaks of the tetraploid samples.

The algorithm for model fitting and selection is implemented in fitTetra, an R package [21] which is included as Additional files 1 and 2. FitTetra produces output in tabular form, including (1) a specification of the fitted model with a.o. the means and mixing proportions of the mixture components, and (2) a list of samples, their probabilities of belonging to each of the mixture components and their assigned genotypes. Further it produces a graphical presentation with a histogram of the allele signal ratio distribution, the fitted model and the genotypes assigned to the samples. If data on diploid samples are also available, a histogram with the signal ratios of these samples is shown superimposed on the tetraploid histogram for visual comparison; the diploid samples are not used in the model fitting or selection. A typical example is shown in Figure 1.

## Results

### Selection of useful SNPs

The GoldenGate data set consisted of 384 SNPs that were scored on 224 tetraploid samples, resulting in 86016 data points of which 70556 reached the signal intensity threshold. Sixty-three of the 384 SNPs (16%) were rejected because less than 60% of the samples reached this threshold.

**Figure 2:**
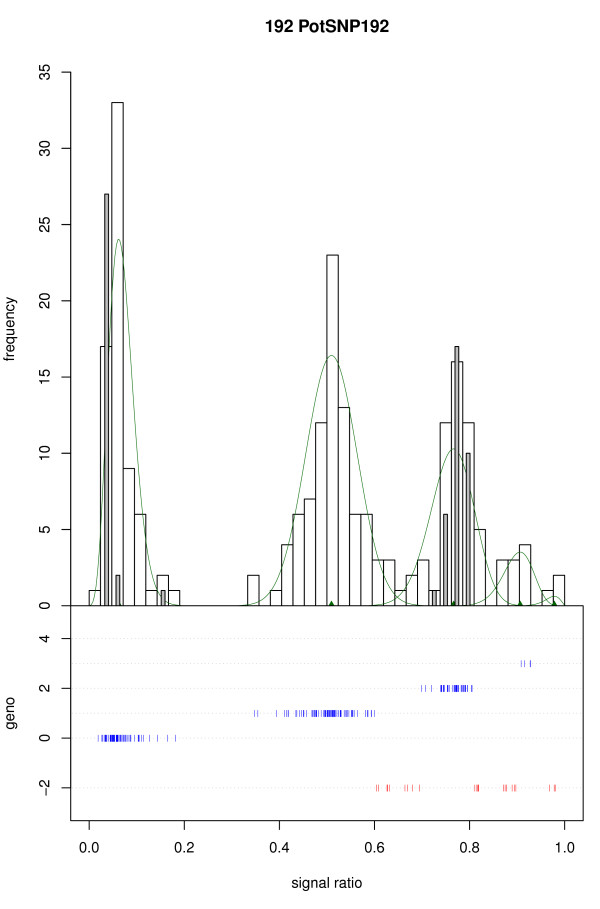
**SNP showing a large gap**. For this SNP (PotSNP192) the signal strength of the b allele is smaller than that of the a allele, resulting in a non-central position of the duplex peak and a wide gap between the nulliplex and simplex peak. For a general explanation see Figure 1.

In the first step of the model selection, 9 of the 321 SNPs were rejected because model fitting failed for numerical reasons with all of the 8 models and both start configurations used for the component means. Visual inspection showed that 7 of these 9 SNPs had no clear peak pattern and 2 were monomorphic. Of the remaining 312 SNPs, in 58 cases one of the five component distributions appeared to be fitted in a wide gap in the histogram; in these cases the second step of model fitting was performed with an adjusted starting configuration of means, which in 45 cases resulted in an improved fit (Figure 2).

Finally, of the 312 SNPs 74 were discarded because less than 60% of the samples could be assigned a genotype, 26 because more than 85% of the samples were scored in one peak and 4 because the standard deviation of the component distributions was above the threshold (0.1 on the transformed scale), leaving 208 SNPs that delivered genotyping data useful for allele dose determination.

Visual inspection showed that of the 26 SNPs that were discarded because more than 85% of the samples was in one peak, 15 were completely monomorphic, while in 11 cases a small number of samples was found outside the peak. The other rejected SNPs all showed an unclear, diffuse pattern in the signal ratio histograms.

Of the 208 SNPs with genotype scores, on visual inspection nine were dominated by one large peak. While the large peak contained less than 85% of the samples and the SNP was therefore not rejected, the remaining samples for these SNPs did not show clear peaks and their scoring seemed uncertain. Also, one SNP (PotSNP234) showed an unclear peak pattern with apparently a small simplex peak between larger nulliplex and duplex peaks. When this interpretation of the peaks is correct, the fitted model and most assigned genotypes for this SNP are incorrect, as the fitted simplex peak rather than the duplex peak coincides with the heterozygous diploid peak. Another SNP (PotSNP373) showed a highly unequal signal intensity for both alleles, with the mean of the duplex peak above 0.85; also in this case the fitted model and most assigned genotypes were incorrect.

### Validation and application of the SNPs

Generally the presence of diploid samples allows a visual check on the correctness of the fitted mixture model. For 123 of the 206 fitted SNPs (excluding the incorrectly fitted PotSNP234 and PotSNP373) the diploid samples were polymorphic. In 110 of these, the diploid peaks coincided with the nulliplex, duplex and quadruplex peaks of the tetraploid varieties. In 13 cases the positions of the diploid peaks did not match that of the corresponding tetraploid peaks.

**Figure 3:**
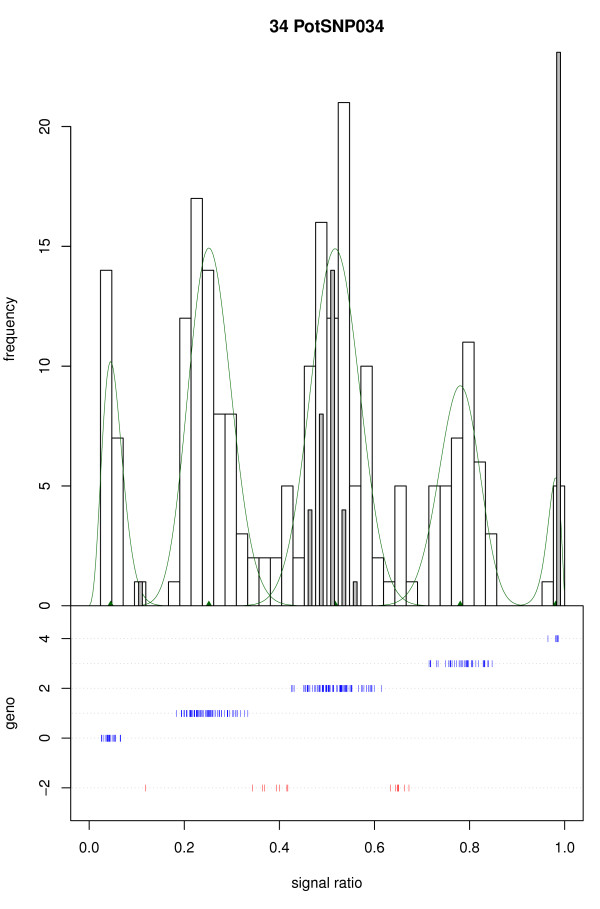
**SNP with possible null alleles**. The possible extra peaks observed between the simplex and duplex, and between the duplex and triplex peak may indicate the presence of null alleles for the SNP; this is not detected by the software. For a general explanation see Figure 1.

The presence of null alleles may be indicated by the presence of extra peaks between the duplex and the simplex and/or triplex peaks. While such peaks can be observed visually in several histograms (e.g. Figure 3, PotSNP034) it is not clear whether these really represent aab0 or abb0 genotypes, or are just a random phenomenon. We have tried to test for the presence of an excess of samples between the scored duplex and simplex or triplex samples based on the fitted mixture model, but this did not produce conclusive results. Therefore fitTetra cannot give an indication of the possible presence of null alleles.

For the 208 fitted SNPs a total of 45702 data points with a signal above the threshold level were available. Of these data points, 40392 (88.4%) were assigned a genotype, as the probability of belonging to any of the 5 classes was above 0.99. The percentage of assigned genotypes varied between SNPs from 62.3% to 100.0%. Among the 208 selected models, the mixture component means were constrained in 56 cases according to Model 1, in 114 cases to Model 2, in 15 cases to Model 3 and in 23 cases to Model 4. In 195 out of the 208 models (93.8%) the mixing proportions were constrained according to HWE ratios, meaning that the observed frequencies of the five genotype classes over all samples were close to those expected under HWE. The 13 SNPs for which non-HWE models were selected included eight that were dominated by one large peak, and the two incorrectly fitted SNPs. This left three SNPs (PotSNP006, PotSNP131, PotSNP184) with a regular, fully informative distribution with a non-HWE model.

## Discussion

### Assigning potato varieties to a genotype class

In this paper we describe the development of fitTetra, an R package that assigns genotype scores to tetraploid samples for bi-allelic markers. We evaluated fitTetra using data for 384 SNPs obtained using the GoldenGate technology for a collection 224 potato varieties. Not all SNPs turned out to be equally suited for assigning a genotype score in this collection. We took the approach that it is better to reject uncertain scores and low-quality SNPs than to try to assign all samples a score. For the GoldenGate array this resulted in the selection of 208 out of the 384 SNPs, i.e. 54%. For the selected SNPs, overall about 88% of the samples were assigned a genotype.

These statistics depend on the choice of the thresholds applied during model selection and the assigning of genotypes. We have shown that the default settings, applied in the current study, perform quite well: the rejected SNPs are either (almost) monomorphic or do not show a clear peak pattern based on visual evaluation, and the samples not assigned a genotype are always in the area between neighbouring peaks. However, these thresholds can be adjusted by the users to fit their specific data sets. The visual inspection of the output data should be done on a representative sample of the markers to set the various options to suitable values for the data set under evaluation.

Validating the genotype assignments in the varieties in an independent way is not easy as there is no 'gold standard'. Therefore we used the position of the assignments in a diploid mapping population as a reference. In 193 of the 206 SNPs (excluding two evidently mis-fitted SNPs) the distribution of the diploid peaks matched that of the tetraploid peaks. In only 13 SNPs (6.3%) this was not the case. This was not due to a mis-assignment of the tetraploid peaks, but rather to a difference in the intrinsic X and/or Y signal strengths between the tetraploid varieties and the diploid population. The reason for this remains unclear, but might be related to SNPs close to the interrogated SNP that interfere with the assay and result in a lower signal in the diploid material. Alternatively also (partial) amplification of paralogous sequences may explain the observation. As the diploid samples were derived from two semi-wild parents [22] and the SNP assays were based on ESTs from tetraploid varieties, the diploid population might harbour such additional SNPs or different paralogous sequences not present in the tetraploid varieties. We attempted to test this assumption by blasting the GoldenGate sequences of these 13 SNPs against the sequences of the RH parent (EMBL, November 2010). However we found only two hits, in one of which additional SNPs were present; which is not sufficient to allow a general conclusion.

SNPs that are not selected for assigning genotype scores in the tetraploid variety panel are not necessarily unusable in other contexts. For instance 44 of the 176 rejected SNPs (25%) could be mapped with high confidence in the diploid SH × RH cross progeny [23]. The most likely reason for performing differently in a wide range of germplasm compared to a well-defined mapping population might be that there are SNPs in the region of the interrogated SNP that interfere with the assay.

### Comparison between fitTetra and beadarrayMSV

We compared fitTetra with the recently published package beadarrayMSV [14]. BeadarrayMSV is designed to analyse SNPs in duplicated loci or (partially) tetraploid species with disomic inheritance. Like in our autotetraploid (potato) case five different allele ratios are possible in these situations. However, as described by [14] several different segregation patterns are possible in such a situation, different from the patterns occurring in an autotetraploid. As fitTetra and beadarrayMSV were developed to analyse the patterns observed in these different situations it is not very surprising that they perform (considerably) less well with data sets of the other type, as described in Additional file 3.

### Application of the approach

The genotype scores can be applied first of all to improve genetic studies in tetraploids. Classical mapping can be carried out more efficiently when all markers that segregate can be used and not just only the nulliplex and simplex markers. For association mapping one could take into account the allele dose, which might result in a more precise estimate of the linkage disequilibrium. SNP markers will also be useful in variety identification in polyploids [24-26]. In this context the genotype scores can improve the resolving power of the markers.

An interesting observation from our analysis of a large collection of tetraploid potato varieties is that almost none of studied markers show evidence against HWE ratios. In general HWE results from random mating in a population. One might expect that potato breeding involves non-random selection of cross parents and cross progeny. While this selection may have resulted in an overall shift of allele frequencies at certain loci it generally does not seem to result in a departure from HWE genotype ratios among varieties.

The use of the approach and the package is not restricted to data obtained from GoldenGate experiments. In principle it can be applied to data that are obtained with any bi-allelic marker system that produces different signals that are proportional to the allele dose. Thus we expect the system to work also well for Infinium, Fluidigm or KASPar http://www.kbioscience.co.uk/ derived data from (auto)tetraploid species. In addition it will also be useful for the analysis of Pyrosequencing data [27], where intensity data per allele are obtained that can be transformed into genotype scores. Finally, while fitTetra is specific for tetrasomically inherited markers, the approach can in principle be generalized to other ploidy levels; how well this will work depends mainly on the noise level of the data, as additional and more closely spaced peaks will be present at higher ploidy levels.

## Conclusions

Until now automated SNP genotype calling in tetraploid species was not possible, which hampered genetic analysis. We have developed and evaluated an R package called fitTetra, that efficiently assigns genotype scores to bi-allelic markers in tetraploid species. The package can in principle be used for any type of bi-allelic marker, including Golden Gate, Infinium and Kaspar, and any tetraploid species.

## Data set

The GoldenGate data set was obtained using the Illumina GoldenGate array with 384 SNPs, as described by [23]. A collection of 224 tetraploid potato varieties covering a wide variation with respect to geographic origin, year of first registration and intended application (fresh consumption, chips, crisps, starch production) was genotyped using this array. Variety codes the tetraploid data set refer to the varieties as described in [6]. In addition 64 diploid samples were analyzed, 58 of which were a subset of the SHxRH cross population [22,23]. The actual genotyping was performed by Service XS Leiden, The Netherlands as described in [23].

From the output of the assay we used the Raw_X and Raw_Y columns to calculate a total signal intensity (the square root of the sum of Raw_X squared and Raw_Y squared) and an allele signal ratio (Raw_X divided by the sum of Raw_X and Raw_Y). A histogram of the signal intensities revealed a peak of low-intensity observations, separated from the higher-intensity observations by a dip around 3200 (not shown). Therefore we removed all observations with a total signal intensity < 3200. The calculated signal ratios together with the SNP_Name and Sample_ID columns were then used as input for our algorithm, after splitting the data into a tetraploid data set (for model fitting) and a diploid data set (for plotting the diploid histograms superimposed on the tetraploid models). The tetraploid and diploid data sets are included in the fitTetra package (Additional file 1).

## Availability and requirements

• Project name: fitTetra

• Project home page: http://www.plantbreeding.wur.nl/UK/software_fitTetra.html

• Operating system(s): Any platform for which the R software [21] is implemented, including Microsoft Windows and Linux. A version compiled for Windows is included as Additional file 2.

• Programming language: R [21]. The package requires R version 2.12.1 or newer; this is relevant only for the Windows 32-bit implementation of R which contained an error in some earlier versions.

• Other requirements: None.

• License: GNU General Public License.

• Any restrictions to use by non-academics: None.

## Authors' contributions

REV developed the model selection algorithm and drafted the manuscript. GG implemented the fitting of mixture models using the EM algorithm. REV and GG together investigated and evaluated various possible models. BV initiated the project in which the data set was obtained and the study leading to this manuscript. All three authors wrote sections of the manuscript and contributed to the discussion. All authors read and approved the final manuscript.

## Supplementary Material

Additional file 1 **The fitTetra R package**. Additional file 1: "fitTetra_1.0.tar.gz" contains the R package fitTetra described in this article, performing the mixture model fitting and model selection. It contains the tetraploid and diploid data used in this article and includes detailed help pages describing the use, input and output of the three user functions. FitTetra is distributed under the GNU Public License http://www.gnu.org/ and is also available from http://www.plantbreeding.wur.nl/UK/software.html. Note that the downloaded file should be renamed to "fitTetra_1.0.tar.gz" before installing the package. Click here for file

Additional file 2 **A compiled version of the fitTetra R package**. Additional file 2: "fitTetra_1.0.zip" contains the fitTetra package compiled for the Windows operating system. Note that the downloaded file should be renamed to "fitTetra_1.0.zip" before installing the package. Click here for file

Additional file 3 **Comparison of fitTetra and beadarrayMSV**. Additional file 3 "Comparison of fitTetra and beadarrayMSV.pdf"describes the comparison that was made between fitTetra and beadarrayMSV, using the potato data from this article and the salmon data from [14]. Click here for file
