# Supplementary material for: Genotype calling in tetraploid species from bi-allelic marker data using mixture models
Source: BMC Bioinformatics. 2011 May 19;12:172. doi: 10.1186/1471-2105-12-172 (PMC3121645; doi:10.1186/1471-2105-12-172)
Supplement: Additional file 2 — A compiled version of the fitTetra R package. Additional file 2: "fitTetra_1.0.zip" contains the fitTetra package compiled for the Windows operating system. Note that the downloaded file should be renamed to "fitTetra_1.0.zip" before installing the package. [file 1471-2105-12-172-S2.ZIP › fitTetra/html/00Index.html]

R: fitTetra is an R package for assigning tetraploid genotype
scores

# fitTetra is an R package for assigning tetraploid genotype scores


---

## Documentation for package ‘fitTetra’ version 1.0

- DESCRIPTION file.

## Help Pages

|  |  |
| --- | --- |
| fitTetra-package | Fits mixture models for genotype calling in tetraploid species |
| CodomMarker | Function to fit a mixture model to a vector of signal ratios of a single bi-allelic marker. |
| diplo.potato.SNP | SNP data for diploid potato |
| fitTetra | Function to fit multiple mixture models to signal ratios of a single bi-allelic marker. |
| saveMarkerModels | A function to fit mixture models for series of markers and saving information |
| tetra.potato.SNP | SNP data for tetraploid potato |
