# Supplementary material for: Genotype calling in tetraploid species from bi-allelic marker data using mixture models
Source: BMC Bioinformatics. 2011 May 19;12:172. doi: 10.1186/1471-2105-12-172 (PMC3121645; doi:10.1186/1471-2105-12-172)
Supplement: Additional file 3 — Comparison of fitTetra and beadarrayMSV. Additional file 3 "Comparison of fitTetra and beadarrayMSV.pdf"describes the comparison that was made between fitTetra and beadarrayMSV, using the potato data from this article and the salmon data from [14]. [file 1471-2105-12-172-S3.PDF]

Comparison of fitTetra and beadarrayMSV

BeadarrayMSV (Gidskehaug et al, 2011) is designed to analyze SNPs in duplicated loci or (partial) tetraploids with disomic segregation. As in autotetraploids like potato five different allele ratios are possible; however the expected proportions of those ratios are different. In addition beadarrayMSV (bMSV) handles normal disomic segregation.

The performance of fitTetra was compared to bMSV for the first 144 SNPs in our data set (included in R package fitTetra, Additional file 1) as bMSV performs its analyses by default in batches of 144. BeadarrayMSV is designed to work with Illumina's Infinium technology rather than with Golden Gate and the raw data are not directly compatible; therefore we used the normalized polar coordinate data for intensity (R) and angle (Theta) as reported by Illumina's GenomeStudio software for bMSV (included in Additional file 3), and converted these back to normalized X and Y allele signals for fitTetra. Both software packages were set to discard normalized intensity values below 0.25, which corresponded roughly to the threshold of 3200 for the raw intensities (see Data Set). FitTetra was run with the default settings, while for bMSV several settings were used including the default. Conversely, fitTetra and bMSV were tested with the (normalized) data on the 144 salmon SNPs provided with bMSV. In this case again a cutoff for intensity values below 0.25 was applied.

Results

We analysed the normalized data of the first 144 potato SNPs with fitTetra using the default options, and with bMSV using different options including the default, the elimination of the test for HW ratios (as the expected HW ratios in an autotetraploid are different from the cases for duplicated loci in a diploid) and a lower "wSpreadLim" and "rotationLim" (as the default

settings allowed too easy merging of clusters); we discuss here the optimal options among those we tested (hwAlpha=0, wSpreadLim=0.5, rotationLim=0.5, other options as default with largeSample=FALSE). Among these 144 SNPs, 55 were not fitted by fitTetra because they were essentially monomorphic or showed insufficient peak separation. Five of these 55 were classified as “SNP” (disomic segregation) by bMSV, which is an invalid classification for autotetraploids. Of the other 89 SNPs, which were scored by fitTetra, bMSV scored one (PotSNP076) as monomorphic (this SNP was one of those with one large peak), four (PotSNP 003, 090, 099, 118) as SNPs segregating in only one of two duplicated loci (“MSV-a” and “MSV-b”; these four showed an asymmetrical peak pattern due to a skewed allele frequency), 10 as a non-duplicated disomic locus (although 7 of them had 5 clearly defined peaks), 8 as “MSV-5”: segregating in two loci, and 66 were not scored at all (14 of these had less clear peak patterns although based on the diploid data they appeared to be scored correctly by fitTetra).

Conversely using fitTetra with the salmon data supplied with bMSV also yielded somewhat disappointing results. Of those 144 SNPs, 56 were classified by bMSV as failed, monomorphic or “PSV” (polymorphic between but not within two duplicated loci). FitTetra scored two of the failed SNPs apparently correctly and did not score the other 54. bMSV scored 38 SNPs as “SNP” (disomic segregation), a type not expected in autotetraploids. FitTetra did not score 18 of these. Of the remaining 20, 16 were clearly of type “SNP”; these were scored into 3 classes by fitTetra, but usually the allele dosages were incorrect. Four of the markers scored as “SNP” by bMSV might also have been of type “MSV-a” or”MSV-b”; in that case 3 of them were scored correctly by fitTetra. Further, 37 markers were scored by bMSV as “MSV-a” or “MSV–b” (segregating in one of two duplicated loci) of which 9 showed a pattern with only two peaks. FitTetra did not score any of these 9 last markers; of the other 28 it scored 14 of these apparently correctly, misscored 10 of them, and four were

not scored. Finally 13 markers were scored by bMSV as MSV-5 (segregation in both duplicated loci); fitTetra scored 6 of these correctly, misscored 4 and did not score 3.

Overall it was clear that each software package produced the best results with the data set for which it was developed. Concerning performance, the speed of bMSV was much higher than that of fitTetra.

Reference

Gidskehaug L, Kent M, Hayes BJ, Lien S: **Genotype calling and mapping of multisite variants using an Atlantic salmon iSelect SNP array.** *Bioinformatics* 2011, 27: 303-310.
